# Supplementary material for: Virulence Factors in Colorectal Cancer Metagenomes and Association of Microbial Siderophores with Advanced Stages
Source: Microorganisms. 2022 Nov 30;10(12):2365. doi: 10.3390/microorganisms10122365 (PMC9781273; doi:10.3390/microorganisms10122365)
Supplement: Supplementary file 1 [file microorganisms-10-02365-s001.zip › microorganisms-1977311-supplementary/microorganisms-1977311/S3 GO- annotation ( supplemantary data 3.odt]

| Virulence factor | Spearman correlation | Chi-square test | Uniprot (GO) annotation | KEGG pathway annotation |
| --- | --- | --- | --- | --- |
| Normal / Small Adenoma |
| Type 3 Fimbrial Minor Pilin Subunit Mrkf (Klebsiella pneumoniae) | r = 0.22 p =  0.03 | 0.034 | Involved In Cell Adhesion (UniProt : A0A0W8ANM7) | Not included in KEGG Pathway database |
| Fimbrial Adhesin Protein Precursor Mrkd (Klebsiella pneumoniae) | r = 0.22 p =  0.03 | 0.034 | Involved In Cell Adhesion (UniProt :P21648) | Not included in KEGG Pathway database |
| Normal / Large  adenoma |
| Type 1 Fimbriae Regulatory Protein Fime (Escherichia coli CFT073) | r =  -0.23 p =  0.047 | 0.047 | Involved In : DNA Integration DNA Recombination Cellular Response To DNA Damage Stimulus (UniProt : P0ADH7) | Not included in KEGG Pathway database |
| Type 3 Fimbrial Minor Pilin Subunit Mrkf (Klebsiella pneumoniae) | r = 0.23 p =  0.046 | 0.046 | Involved In Cell Adhesion (UniProt : A0A0W8ANM7) | Not included in KEGG Pathway database |
| UTP-glucose-1-phosphate uridylyltransferase subunit GalF (Klebsiella pneumoniae) | r =  0.23 p =  0.046 | 0.046 | Enables : enzyme regulator activity nucleotidyltransferase activity UTP:glucose-1-phosphate uridylyltransferase activity transferase activity Involved in : biosynthetic process UDP-glucose metabolic process lipopolysaccharide biosynthetic process nucleotide-sugar metabolic process regulation of catalytic activity (UniProt : A0A1C3SZT3) | Not included in KEGG Pathway database |
| Normal /  cancer I-II |
| dTDP-glucose 46-dehydratase (Haemophilus influenzae) | r =  0.26 p =  0.018 | 0.019 | Enables : dTDP-glucose 4,6-dehydratase activity lyase activity Involved in : dTDP-rhamnose biosynthetic process O antigen biosynthetic process lipopolysaccharide biosynthetic process extracellular polysaccharide biosynthetic process nucleotide-sugar metabolic process (UniProt : P44914) | Streptomycin biosynthesis ([hip00521](https://www.genome.jp/pathway/hip00521+CGSHiEE_07665)) O-Antigen nucleotide sugar biosynthesis ([hip00541](https://www.genome.jp/pathway/hip00541+CGSHiEE_07665)) Metabolic pathways ([hip01100](https://www.genome.jp/pathway/hip01100+CGSHiEE_07665)) Biosynthesis of secondary metabolites ([hip01110](https://www.genome.jp/pathway/hip01110+CGSHiEE_07665)) Biosynthesis of nucleotide sugars ([hip01250](https://www.genome.jp/pathway/hip01250+CGSHiEE_07665)) |
| 2,3-dihydro-2,3-dihydroxybenzoate synthetase, isochorismatase (Klebsiella pneumoniae) | r =  0.26 p =  0.018 | 0.019 | Enables :  isochorismatase activity (UniProt : A6T642) | Biosynthesis of siderophore group nonribosomal peptides ([kpn01053](https://www.kegg.jp/pathway/kpn01053+KPN_00613) ) Biosynthesis of secondary metabolites ([kpn01110](https://www.kegg.jp/pathway/kpn01110+KPN_00613) ) |
| transcriptional regulator RcsB (Klebsiella pneumoniae) | r =  0.24 p =  0.027 | 0.027 | Enables : DNA binding Involved in : phosphorelay signal transduction system regulation of transcription, DNA-templated (UniProt : A0A0H3GVM1) | Two-component system ([kpm02020](https://www.kegg.jp/pathway/kpm02020+KPHS_37040)) |
| Type 3 Fimbrial Minor Pilin Subunit Mrkf (Klebsiella pneumoniae) | r =  0.26  p =  0.018 | 0.019 | Involved In Cell Adhesion (UniProt : A0A0W8ANM7) | Not included in KEGG Pathway database |
| Fimbrial Adhesin Protein Precursor Mrkd (Klebsiella pneumoniae) | r =  0.26  p =  0.018 | 0.019 | Involved In Cell Adhesion (UniProt :P21648) | Not included in KEGG Pathway database |
| fimbrial biogenesis outer membrane usher protein mrkC precursor (Klebsiella pneumoniae) | r =  0.26  p =  0.018 | 0.019 | Enables :  fimbrial usher porin activity Involved in : pilus assembly transmembrane transport | Not included in KEGG Pathway database |
| Normal /  cancer III-IV |
| Enterobactin Synthase Multienzyme Complex Component, ATP-Dependent (Escherichia coli CFT073) | r =  0.23  p =  0.028 | 0,028 | Enables : Catalytic Activity Transferase Activity Nucleotidyltransferase Activity Ligase Activity phosphopantetheine binding 2,3-dihydroxybenzoate-serine ligase activity ATP binding  Involved In : Secondary Metabolite Biosynthetic Process enterobactin biosynthetic process amino acid activation for nonribosomal peptide biosynthetic process biosynthetic process metabolic process (UniProt : P11454) | Biosynthesis of secondary metabolites ([ecj01110](https://www.kegg.jp/pathway/ecj01110+JW0578)) Biosynthesis of siderophore group nonribosomal peptides ([ecj01053](https://www.kegg.jp/pathway/ecj01053+JW0578)) |
| Type 1 Fimbriae Regulatory Protein Fimb (Escherichia coli CFT073) | r =  0.23 p =  0.024 | 0,024 | Involved In : DNA Integration DNA Recombination Cellular Response To DNA Damage Stimulus (UniProt : P0ADH5) | Not included in KEGG Pathway database |
| Type 1 Fimbriae Regulatory Protein Fime (Escherichia coli CFT073) | r =  0.21 p =  0.037 | 0,037 | Involved In : DNA Integration DNA Recombination Cellular Response To DNA Damage Stimulus (UniProt : P0ADH7) | Not included in KEGG Pathway database |
| Type-1 Fimbrial Protein, A Chain Precursor (Escherichia coli CFT073) | r =  0.32 p =  0.002 | 0,002 | Involved in : Cell Adhesion cell adhesion involved in single-species biofilm formation (UniProt : P04128) | Not included in KEGG Pathway database |
| Tir domain containing protein TcpC (Escherichia coli CFT073) | r =  0.26 p =  0.012 | 0,406 | Enables : NAD+ nucleosidase activity hydrolase activity NAD(P)+ nucleosidase activity Involved in : signal transduction NAD catabolic process negative regulation of MyD88-dependent toll-like receptor signaling pathway (UniProt : A0A0H2V8B5) | Not included in KEGG Pathway database |
| Ferrienterobactin ABC Transporter Atpase (Escherichia coli CFT073) | r =  0.23 p =  0.024 | 0,024 | Enables: ATP Binding Nucleotide Binding  (UniProt : Q8XBV7) | ABC Transporters [ece02010](https://www.kegg.jp/kegg-bin/show_pathway?ece02010) |
| Iron-Enterobactin ABC Transporter Permease (Escherichia coli CFT073) | r =  0.26  p =  0.012 | 0,013 | Involved In Transmembrane Transport (UniProt : A0A061KSK2) | ABC Transporters [ecd02010](https://www.kegg.jp/pathway/ecd02010+ECDH10B_0549) |
| Phosphopantetheinyl transferase component of enterobactin synthase multienzyme complex (Escherichia coli CFT073) | r =  0.267 p =  0.010 | 0,011 | Enables : holo-[acyl-carrier-protein] synthase activity phosphotransferase activity, for other substituted phosphate groups magnesium ion binding transferase activity metal ion binding Involved in : siderophore metabolic process enterobactin biosynthetic process protein phosphopantetheinylation (UniProt : P19925) | Biosynthesis of siderophore group nonribosomal peptides ([ecj01053](https://www.kegg.jp/pathway/ecj01053+JW5085)) Biosynthesis of secondary metabolites ([ecj01110](https://www.kegg.jp/pathway/ecj01110+JW5085)) |
| isochorismate synthase 1 (Escherichia coli CFT073) | r =  0.28 p =  0.007 | 0,008 | Enables : isochorismate synthase activity magnesium ion binding isomerase activity metal ion binding Involved in : biosynthetic process enterobactin biosynthetic process (UniProt : P0AEJ2) | Ubiquinone and other terpenoid-quinone biosynthesis ([ecj00130](https://www.kegg.jp/pathway/ecj00130+JW0585)) Biosynthesis of siderophore group nonribosomal peptides ([ecj01053](https://www.kegg.jp/pathway/ecj01053+JW0585)) Metabolic pathways ([ecj01100](https://www.kegg.jp/pathway/ecj01100+JW0585)) Biosynthesis of secondary metabolites ([ecj01110](https://www.kegg.jp/pathway/ecj01110+JW0585)) |
| 2,3-dihydroxybenzoate-AMP ligase component of enterobactin synthase multienzyme complex (Escherichia coli CFT073) | r =  0.27  p =  0.009 | 0,010 | Enables : 2,3-dihydroxybenzoate-serine ligase activity (2,3-dihydroxybenzoyl)adenylate synthase activity ATP binding transferase activity acyltransferase activity ligase activity protein binding nucleotide binding Involved in : siderophore biosynthetic process enterobactin biosynthetic process (UniProt : P10378) | Biosynthesis of siderophore group nonribosomal peptides ([ecj01053](https://www.kegg.jp/pathway/ecj01053+JW0586)) Biosynthesis of secondary metabolites ([ecj01110](https://www.kegg.jp/pathway/ecj01110+JW0586)) |
| Polymerized tip adhesin of ECP fibers (Escherichia coli O157:H7 str. EDL933) | r =  0.24 p =  0.02 | 0,021 | early-stage biofilm development  host cell recognition (Uniprot : B7UJD8) | Not included in KEGG Pathway database |
| Fimg Protein Precursor (Escherichia coli CFT073) | r =  0.28 p =  0.006 | 0,007 | Involved in : mechanosensory behavior cell-substrate adhesion cell adhesion cell adhesion involved in single-species biofilm formation (UniProt : P08190) | Not included in KEGG Pathway database |
| ferrienterobactin ABC transporter periplasmic binding protein (Escherichia coli CFT073) | r =  0.22 p =  0.033 | 0,033 | Involved in : ion transport iron ion homeostasis iron import into cell Acts upstream of or within : ferric-enterobactin import into cell cellular response to DNA damage stimulus (UniProt : P0AEL6) | ABC transporters ([ecs02010](https://www.kegg.jp/pathway/ecs02010+ECs_0631)) |
| ferrienterobactin ABC transporter permease (Escherichia coli CFT073) | r =  0.28 p =  0.007 | 0,007 | Enables : transmembrane transporter activity (UniProt : C3TK52) | ABC transporters ([ecs02010](https://www.kegg.jp/pathway/ecs02010+ECs_0629)) |
| Outer Membrane Protein A (Escherichia coli O18:K1:H7 str. RS218) | r =  0.25 p =  0.017 | 0,017 | Enables : Porin Activity Identical Protein Binding Protein Binding Ion Transport Involved in : Cellular Response To DNA Damage Stimulus Ion Transport Detection Of Virus Viral Entry Into Host Cell (UniProt : P0A910) | Not included in KEGG Pathway database |
| E. coli common pilus usher EcpC (Escherichia coli O157:H7 str. EDL933) | r =  0.25 p =  0.017 | 0,017 | early-stage biofilm development host cell recognition (UniProt : Q8X6I4) | Not included in KEGG Pathway database |
| Type III secretion system effector espL1 (Escherichia coli O157:H7 str. EDL933) | r =  0.22 p =  0.03 | 0,035 | Not Included In GO database | Not included in KEGG Pathway database |
| Type III secretion system effector EspX1 (Escherichia coli O157:H7 str. EDL933) | r =  0.24 p =  0.022 | 0,022 | No Functional Annotation In Uniprot | Not Included In Pathway |
| enterobactin/ferric enterobactin esterase (Escherichia coli CFT073) | r =  0.24 p =  0.02 | 0,021 | Enables : iron ion binding enterochelin esterase activity hydrolase activity Involved in : iron ion transport  Acts upstream of or within : siderophore-dependent iron import into cell enterobactin catabolic process (UniProt : P13039) | Biosynthesis of siderophore group nonribosomal peptides ([eco01053](https://www.kegg.jp/kegg-bin/show_pathway?eco01053)) |
| Enterobactin Exporter, Iron-Regulated (Escherichia coli CFT073) | r =  0.29 p =  0.005 | 0,006 | Involved in : siderophore transport transmembrane transport enterobactin transport Acts upstream of or within : cellular response to DNA damage stimulus response to antibiotic (UniProt :P24077) | Biosynthesis of siderophore group nonribosomal peptides ([eco01053](https://www.kegg.jp/kegg-bin/show_pathway?eco01053)) |
| general secretion pathway protein D (Shigella dysenteriae) | r =  0.22 p =  0.029 | 0,030 | Involved in : protein secretion by the type II secretion system protein secretion protein transport (UniProt :A0A090NM48) | Bacterial secretion system ([shq03070](https://www.kegg.jp/pathway/shq03070+A0259_18545)) |
| general secretion pathway protein F (Shigella dysenteriae) | r =  0.20 p =  0.048 | 0,048 | Enables : metal ion binding Involved in : protein secretion by the type II secretion system protein secretion protein transport (UniProt : Q32C44) | Bacterial secretion system ([sdy03070](https://www.genome.jp/pathway/sdy03070+SDY_3095)) |
| general secretion pathway protein K (Shigella dysenteriae) | r =  0.21 p =  0.037 | 0,037 | Involved in : protein secretion protein transport (UniProt : B3X6U7) | Bacterial secretion system ([shq03070](https://www.kegg.jp/pathway/shq03070+A0259_18545)) |
| UDP-glucose 6-dehydrogenase (Klebsiella pneumoniae) | r =  0.20 p =  0.049 | 0,048 | Enables : oxidoreductase activity oxidoreductase activity, acting on the CH-OH group of donors, NAD or NADP as acceptor UDP-glucose 6-dehydrogenase activity NAD binding Involved in : polysaccharide biosynthetic process (UniProt : A6TBE0) | Pentose and glucuronate interconversions ([kpn00040](https://www.kegg.jp/pathway/kpn00040+KPN_02493)) Ascorbate and aldarate metabolism ([kpn00053](https://www.kegg.jp/pathway/kpn00053+KPN_02493)) Amino sugar and nucleotide sugar metabolism ([kpn00520](https://www.kegg.jp/pathway/kpn00520+KPN_02493)) O-Antigen nucleotide sugar biosynthesis ([kpn00541](https://www.kegg.jp/pathway/kpn00541+KPN_02493)) Metabolic pathways ([kpn01100](https://www.kegg.jp/pathway/kpn01100+KPN_02493)) Biosynthesis of cofactors ([kpn01240](https://www.kegg.jp/pathway/kpn01240+KPN_02493)) Biosynthesis of nucleotide sugars ([kpn01250](https://www.kegg.jp/pathway/kpn01250+KPN_02493)) |
| UTP-glucose-1-phosphate uridylyltransferase subunit GalF (Klebsiella pneumoniae) | r =  0.20 p =  0.049 | 0,048 | Enables : nucleotidyltransferase activity enzyme regulator activity nucleotidyltransferase activity UTP:glucose-1-phosphate uridylyltransferase activity Involved in : regulation of catalytic activity nucleotide-sugar metabolic process biosynthetic process UDP-glucose metabolic process lipopolysaccharide biosynthetic process (UniProt : W9BIC5) | Pentose and glucuronate interconversions ([kpnk00040](https://www.kegg.jp/pathway/kpnk00040+BN49_3731)) Galactose metabolism ([kpnk00052](https://www.kegg.jp/pathway/kpnk00052+BN49_3731)) Starch and sucrose metabolism ([kpnk00500](https://www.kegg.jp/pathway/kpnk00500+BN49_3731)) Amino sugar and nucleotide sugar metabolism ([kpnk00520](https://www.kegg.jp/pathway/kpnk00520+BN49_3731)) O-Antigen nucleotide sugar biosynthesis ([kpnk00541](https://www.kegg.jp/pathway/kpnk00541+BN49_3731)) Metabolic pathways ([kpnk01100](https://www.kegg.jp/pathway/kpnk01100+BN49_3731)) Biosynthesis of secondary metabolites ([kpnk01110](https://www.kegg.jp/pathway/kpnk01110+BN49_3731)) Biosynthesis of cofactors ([kpn01240](https://www.kegg.jp/pathway/kpn01240+KPN_02493)) Biosynthesis of nucleotide sugars ([kpn01250](https://www.kegg.jp/pathway/kpn01250+KPN_02493)) |
| Yersiniabactin Siderophore Biosynthetic Protein (Yersinia pestis) | r =  0.24 p =  0.022 | 0,022 | Enables : ligase activity Involved in : negative regulation of transcription by RNA polymerase II negative regulation of transcription, DNA-templated (UniProt : Q56950) | Not included in KEGG Pathway database |
| Aerobactin Synthesis Protein Iucb (Shigella flexneri) | r =  0.23 p =  0.026 | 0,026 | Enables : transferase activity acyltransferase activity Involved in : siderophore biosynthetic process (UniProt : A0A3Y2RUB6) | Biosynthesis of various other secondary metabolites (sfx00997  ) Metabolic pathways (sfx01100) Biosynthesis of secondary metabolites (sfx01110) Microbial metabolism in diverse environments (sfx01120) |
| Putative Signal Transducer (Yersinia pestis) | r =  0.20 p =  0.048 | 0,048 | Enables : transmembrane transporter activity (UniProt : A0A380PD93) | Not included in KEGG Pathway database |
| yersiniabactin receptor FyuA (Klebsiella pneumoniae) | r =  0.22 p =  0.032 | 0,032 | Enables : siderophore uptake transmembrane transporter activity signaling receptor activity Involved in : siderophore transport siderophore transmembrane transport (UniProt : A0A483IZD4) |  |
| Small adenoma / Large adenoma |
| no significant correlation |
| Large adenoma / cancer I-II |
| no significant correlation |
| Adenoma / cancer I-II |
| 2,3-dihydro-2,3-dihydroxybenzoate synthetase, isochorismatase entB (Klebsiella pneumoniae) | r =  0.24 p =  0.048 | 0.047 | Enables :  isochorismatase activity (UniProt : A6T642) | Biosynthesis of siderophore group nonribosomal peptides ([kpn01053](https://www.kegg.jp/pathway/kpn01053+KPN_00613) ) Biosynthesis of secondary metabolites ([kpn01110](https://www.kegg.jp/pathway/kpn01110+KPN_00613) ) |
| type vi secretion system contractile sheath small subunit vipa (Klebsiella pneumoniae) | r =  0.24 p =  0.048 | 0.047 | Not Included In GO database | Not included in KEGG Pathway database |
| transcriptional regulator rcsb (Klebsiella pneumoniae) | r =  0.30 p =  0.013 | 0.014 | Enables : DNA binding Involved in : phosphorelay signal transduction system regulation of transcription, DNA-templated (UniProt : A0A0H3GVM1) | Two-component system ([kpm02020](https://www.kegg.jp/pathway/kpm02020+KPHS_37040)) |
| polysaccharide lyase, family 8 (Enterococcus faecalis) | r =  0.30 p =  0.013 | 0.014 | Enables : lyase activity carbohydrate binding catalytic activity Involved in : carbohydrate metabolic process (UniProt : Q82ZM8 ) | Not included in KEGG Pathway database |
| cytotoxic necrotizing factor 1 (Escherichia coli str. E-B35) | r =  0.24 p =  0.048 | 0.047 | Bacterial toxins (kegg Brite : [eci02042](https://www.kegg.jp/brite/eci02042+UTI89_C4921)) | Not included in KEGG Pathway database |
| s fimbriae minor subunit sfag (Escherichia coli UTI89) | r =  0.24 p =  0.048 | 0.047 | Involved in : cell adhesion (UniProt : Q1RDG7) | Not included in KEGG Pathway database |
| s fimbriae minor subunit sfas (Escherichia coli UTI89) | r =  0.24 p =  0.048 | 0.047 | Involved in : cell adhesion (UniProt : [Q1RDG6](https://www.uniprot.org/uniprot/Q1RDG6)) | Not included in KEGG Pathway database |
| tir domain containing protein tcpc (Escherichia coli CFT073) | r = 0.30 p = 0.013 | 0.014 | Enables : NAD+ nucleosidase activity hydrolase activity NAD(P)+ nucleosidase activity Involved in : signal transduction NAD catabolic process negative regulation of MyD88-dependent toll-like receptor signaling pathway (UniProt : A0A0H2V8B5) | Not included in KEGG Pathway database |
| cancer I-II / cancer III-IV |
| Type 1 Fimbriae Regulatory Protein Fimb (Escherichia coli CFT073) | r = 0.27 p =  0.04 | 0.046 | Involved In : DNA Integration DNA Recombination Cellular Response To DNA Damage Stimulus (UniProt : P0ADH5) | Not included in KEGG Pathway database |
| Type 1 Fimbriae Regulatory Protein Fime (Escherichia coli CFT073) | r = 0.32 p =  0.019 | 0.019 | Involved In : DNA Integration DNA Recombination Cellular Response To DNA Damage Stimulus (UniProt : P0ADH7) | Not included in KEGG Pathway database |
| Type-1 Fimbrial Protein, A Chain Precursor (Escherichia coli CFT073) | r = 0.33 p =  0.012 | 0.013 | Involved in : Cell Adhesion cell adhesion involved in single-species biofilm formation (UniProt : P04128) | Not included in KEGG Pathway database |
| Fimbrin-Like Protein Fimi Precursor (Escherichia coli CFT073) | r = 0.32 p =  0.019 | 0.019 | Involved in : Cell Adhesion cell adhesion involved in single-species biofilm formation (UniProt : P39264) | Not included in KEGG Pathway database |
| Putative Heme-Binding Protein Chux (Escherichia coli CFT073) | r = 0.27 p =  0.046 | 0.046 | Not Included In GO database | Not included in KEGG Pathway database |
| Ferrienterobactin ABC Transporter Atpase (Escherichia coli CFT073) | r = 0.27 p =  0.047 | 0.046 | Enables: ATP Binding Nucleotide Binding  (UniProt : Q8XBV7) | ABC Transporters [ece02010](https://www.kegg.jp/kegg-bin/show_pathway?ece02010) |
| Iron-Enterobactin ABC Transporter Permease (Escherichia coli CFT073) | r = 0.29 p =  0.034 | 0.034 | Involved In Transmembrane Transport (UniProt : A0A061KSK2) | ABC Transporters [ecd02010](https://www.kegg.jp/pathway/ecd02010+ECDH10B_0549) |
| Transcriptional Regulator Ybta (Yersinia pestis) | r = 0.27 p =  0.046 | 0.046 | Enables : DNA-binding transcription factor activity sequence-specific DNA binding Involved in : cellular response to iron ion regulation of transcription, DNA-templated (UniProt : Q56951) | Not included in KEGG Pathway database |
| Fimg Protein Precursor (Escherichia coli CFT073) | r = 0.39 p =  0.003 | 0.004 | Involved in : mechanosensory behavior cell-substrate adhesion cell adhesion cell adhesion involved in single-species biofilm formation (UniProt : P08190) | Not included in KEGG Pathway database |
| Outer Membrane Protein A (Escherichia coli O18:K1:H7 str. RS218) | r = 0.29 p =  0.034 | 0.121 | Enables : Porin Activity Identical Protein Binding Protein Binding Peptidoglycan Binding Ion Transmembrane Transporter Activity Acts Upstream Of Or Within: Cellular Response To DNA Damage Stimulus Ion Transport Detection Of Virus Viral Entry Into Host Cell (UniProt : P0A910) | Not included in KEGG Pathway database |
| E. Coli Common Pilus Structural Subunit Ecpa (Escherichia coli O157:H7 str. EDL933) | r = 0.29 p =  0.034 | 0.034 | early-stage biofilm development host cell recognition (UniProt : B7UJE1) | Not included in KEGG Pathway database |
| Enterobactin Exporter, Iron-Regulated (Escherichia coli CFT073) | r = 0.32 p =  0.019 | 0.019 | Involved in : siderophore transport transmembrane transport enterobactin transport Acts upstream of or within : cellular response to DNA damage stimulus response to antibiotic (UniProt : P24077) | Biosynthesis of siderophore group nonribosomal peptides [eco01053](https://www.kegg.jp/kegg-bin/show_pathway?eco01053) |
| Enterococcus Faecalis Surface Protein Fss2, Fibrinogen Binding Protein (Enterococcus faecalis V583) | r = -0.29 p =  0.034 | 0.034 | Not Included In GO database | Not included in KEGG Pathway database |
| Heat-Stable Enterotoxin 1 (Escherichia coli O44:H18 042) | r = 0.33 p =  0.013 | 0.014 | Enables : toxin activity Involved in : modulation of process of other organism (UniProt : A0A650FQK3) | Not included in KEGG Pathway database |
| Yersiniabactin Siderophore Biosynthetic Protein (Yersinia pestis CO92) | r = 0.27 p =  0.046 | 0.046 | Enables : ligase activity Involved in : negative regulation of transcription by RNA polymerase II negative regulation of transcription, DNA-templated (UniProt : Q56950) | Not included in KEGG Pathway database |
| Yersiniabactin ABC Transporter ATP-Binding/Permease Protein Ybtp (Klebsiella pneumoniae subsp. pneumoniae NTUH-K2044) | r = 0.30 p =  0.027 | 0.028 | Enables : ATP binding ABC-type transporter activity nucleotide binding hydrolase activity Involved in : transmembrane transport (UniProt : A4GZC9) | Not included in KEGG Pathway database |
| Aerobactin Synthesis Protein Iucb (Shigella flexneri 2a str. 301) | r = 0.27 p =  0.043 | 0.042 | Enables : transferase activity acyltransferase activity acyltransferase activity Involved in : siderophore biosynthetic process (UniProt : A0A3Y2RUB6) | Biosynthesis of various other secondary metabolites (sfx00997  ) Metabolic pathways (sfx01100) Biosynthesis of secondary metabolites (sfx01110) Microbial metabolism in diverse environments (sfx01120) |
| Yersiniabactin Biosynthetic Protein Irp1 (Yersinia pestis CO92) | r = 0.33 p =  0.015 | 0.016 | Enables : fatty acid synthase activity phosphopantetheine binding transferase activity acyltransferase activity 3-oxoacyl-[acyl-carrier-protein] synthase activity catalytic activity transferase activity acyltransferase activity Involved in : fatty acid biosynthetic process secondary metabolite biosynthetic process organic substance biosynthetic process fatty acid biosynthetic process DIM/DIP cell wall layer assembly biosynthetic process  (UniProt : Q9Z373) | Biosynthesis of siderophore group nonribosomal peptides ([ype01053](https://www.genome.jp/pathway/ype01053+YPO1910)) |
| Yersiniabactin Biosynthetic Protein Irp2 (Yersinia pestis CO92) | r = 0.33 p =  0.015 | 0.016 | Enables : phosphopantetheine binding catalytic activity Involved in : organic substance biosynthetic process (UniProt : A0A3N4B635) | Biosynthesis of siderophore group nonribosomal peptides (ype01053) |
| Putative Signal Transducer (Yersinia pestis CO92) | r = 0.30 p =  0.027 | 0.028 | Enables : transmembrane transporter activity (UniProt : A0A380PD93) | Not included in KEGG Pathway database |
| Salicylate Synthase Irp9 (Yersinia pestis CO92) | r = 0.27 p =  0.046 | 0.046 | Enables : oxo-acid-lyase activity isochorismate synthase activity Involved in : cellular biosynthetic process organic substance biosynthetic process cellular response to iron ion tryptophan biosynthetic process biosynthetic process (UniProt: [Q9Z396](https://www.uniprot.org/uniprot/Q9Z396)) | Biosynthesis of siderophore group nonribosomal peptides ([ype01053](https://www.genome.jp/pathway/ype01053+YPO1916)) Biosynthesis of secondary metabolites ([ype01110](https://www.genome.jp/pathway/ype01110+YPO1916)) |
| Colibactin Hybrid Non-Ribosomal Peptide Synthetase/Type I Polyketide Synthase Clbk (Klebsiella pneumoniae subsp. pneumoniae 1084) | r = 0.30 p =  0.028 | 0.028 | Enables : ligase activity phosphopantetheine binding oxidoreductase activity acyltransferase activity catalytic activity transferase activity acyltransferase activity Involved in : organic substance biosynthetic process (UniProt: A0A422Z4P8) | Not included in KEGG Pathway database |
| Colibactin Biosynthesis Dehydrogenase Clbf (Klebsiella pneumoniae subsp. pneumoniae 1084) | r = 0.27 p =  0.048 | 0.048 | Enables : oxidoreductase activity, acting on the CH-CH group of donors flavin adenine dinucleotide binding oxidoreductase activity butyryl-CoA dehydrogenase activity (UniProt: A0A1C1IWF7) | Not included in KEGG Pathway database |
| Colibactin Polyketide Synthase Clbi (Klebsiella pneumoniae subsp. pneumoniae 1084) | r = 0.27 p =  0.048 | 0.048 | Not Included In GO database | Not included in KEGG Pathway database |
| Colibactin Biosynthesis Amidase Clbl (Klebsiella pneumoniae subsp. pneumoniae 1084) | r = 0.27 p =  0.048 | 0.048 | Enables : ligase activity (UniProt: A0A1C1IWG4) | Not included in KEGG Pathway database |
